# Progress in the preparation of an active immunization model of anti-N-methyl-D-aspartate receptor encephalitis in animals

**DOI:** 10.3389/fimmu.2025.1585353

**Published:** 2025-12-10

**Authors:** Kuikui Zeng, Yuting Liu, Sai Yang, Lingjuan Liu

**Affiliations:** 1 Department of Pediatrics, The Second Xiangya Hospital, Central South University, Changsha, Hunan, China; 2 Pediatrics Research Institute, Hunan Children’s Hospital, Central South University, Changsha, Hunan, China; 3 Department of Neurology, Hunan Children’s Hospital, Central South University, Changsha, Hunan, China

**Keywords:** anti-NMDAR encephalitis, animal model, mouse, active immunization, GluN1 subunits, receptor

## Abstract

Anti-N-methyl-D-aspartate receptor (NMDAR) encephalitis is the most common form of autoimmune encephalitis, and its primary immunologic mechanism is that NMDA antibodies bind to the NMDA receptor, causing NMDAR to internalize and weaken to trigger autoimmune encephalitis. Currently, NMDAR antibodies are detected in the cerebrospinal fluid of patients with NMDAR encephalitis, which is associated with autoantibodies against neurosurface or synaptic antigens, and this antibody is an important marker for clinical diagnosis. However, both the complete pathogenesis and the pathophysiological mechanisms of anti-NMDAR encephalitis remain poorly understood. Animal models have been established as a fundamental research strategy to elucidate the underlying mechanisms of this disease. Among these animal models, active immunity models have attracted significant attention due to its superior ability to recapitulate the disease onset and progression. Active immunity models of NMDAR encephalitis contain several classifications, each of which has its specific strengths and scope of applicability, depending on the research objectives. The primary objective of this review is to systematically classify existing active immunization models of anti-NMDAR encephalitis, with detailed discussion on their establishment protocols, respective advantages, current applications, and future prospects. This comprehensive analysis aims to provide valuable references and guidance for future basic research and clinical investigations on NMDAR encephalitis.

## 1 Introduction

In recent years, anti-N-methyl-D-aspartate receptor (NMDAR) encephalitis has been gradually recognized in clinical practice, with its diagnostic rate showing continued improvement. Anti-NMDAR encephalitis is the most common type of autoimmune encephalitis involving the central nervous system (CNS). Most cases are mediated by anti-NMDAR antibodies, with cerebrospinal fluid (CSF) IgG-class anti-NMDAR antibodies serving as the most critical diagnostic biomarker due to their high specificity for confirming the disease (1, 2). Serologic antibody testing may also provide supplementary diagnostic value (3). Anti-NMDAR encephalitis can involve all age groups, and epidemiological characteristics show a predominance of children and youth, among whom females constitute the majority (4, 5). In a retrospective analysis, about 70% of patients presented with prodromal symptoms of infection, such as lethargy, headache, upper respiratory symptoms, fever, etc. (6). It primarily affects the central nervous system, commonly manifesting as neuropsychiatric symptoms, epileptic seizures, memory impairment, sleep disturbances, as well as language and motor dysfunction (2–4, 7–10). Since the 1990s, there has been a growing body of literature on anti-NMDAR encephalitis. Current mechanistic research primarily focuses on the specific role of GluN1 antibodies. These antibodies target extracellular epitopes of the GluN1 subunit of NMDARs, particularly in cortical and hippocampal GABAergic neurons (11). Through cross-linking with NMDARs, they promote receptor internalization, reduce surface receptor density, and ultimately lead to neuronal dysfunction (12). In recent years, with increasing research, more and more animal models have been used to explore the molecular mechanisms of the disease. Due to the short growth cycle and accessibility of mice, the current animal models for autoimmune encephalitis research are all mouse models. Anti-NMDAR animal models can be classified into active immunization models and passive immunization models based on differences in the substances injected and the induced immune mechanisms. The former involves either peptide fragment injection (10, 13) or viral infection (14) to trigger active immunity in mice, while the latter directly administers purified antibodies, lymphocytes, or their derivatives from patients or other mice into animals to confer immune reactivity (15). To date, the precise pathogenesis, underlying pathophysiological mechanisms, and role of inflammatory mediators in anti-NMDAR encephalitis have not been fully elucidated, and currently blocking the immune response is an important method of treatment for this disease, as the establishment of animal models needs to be further optimized, and the pathogenesis of the disease needs to be further verified, so a strong animal model will help to solve these problems.

Therefore, this review systematically summarizes current methodologies for establishing active immunization animal models of anti-NMDAR encephalitis, critically evaluates the advantages and limitations of various modeling approaches, and provides a conceptual framework for future investigations into the precise molecular mechanisms underlying this disorder.

## 2 Introduction to the classification and preparation of NMDAR active immunization animal models

**Figure 1:**
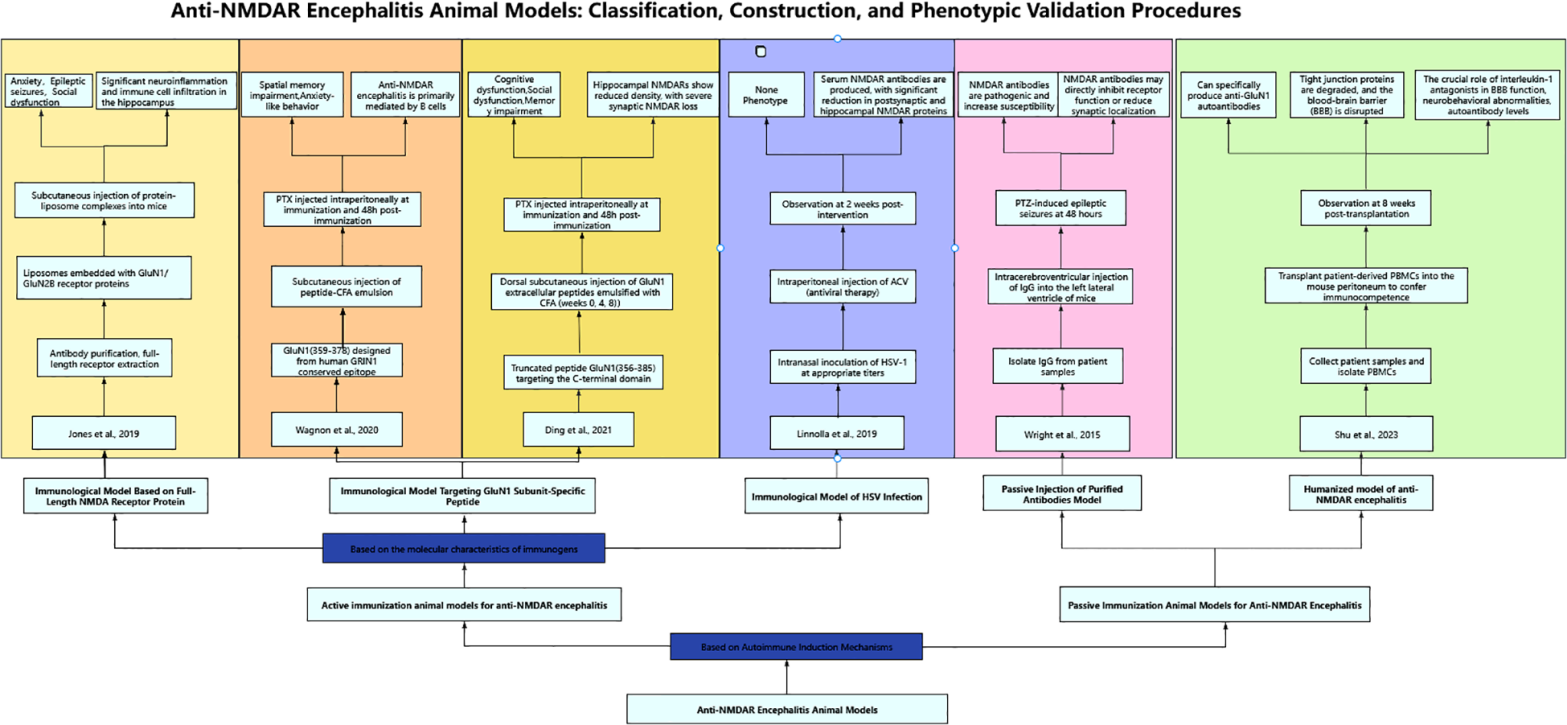
Anti-NMDAR encephalitis animal models: classification, construction, and phenotypic validation procedures.

In current studies scholars have successfully established various active immunization animal models for anti-NMDAR encephalitis. Based on the molecular characteristics of immunogens, these models can be systematically categorized into three types (**Figure 1**): immunization models using complete NMDA receptor proteins, those targeting GluN1 subunit-specific peptides, and herpes simplex virus (HSV) infection-induced immunization models. The following presents a systematic review of the establishment methods, mechanisms of action, and research progress of these models.

### 2.1 Immunological model based on full-length NMDA receptor protein

Both clinical observations of anti-NMDAR encephalitis patients and fundamental studies establishing mouse models have demonstrated that NMDAR antibodies cause: reduction in NMDA receptor density, impairment of receptor-mediated synaptic transmission (3, 16), and decreased surface clustering of NMDAR proteins (17). These antibody-mediated effects ultimately result in diminished glutamate-evoked currents (18, 19). Structurally, the NMDA receptor forms a heterotetrameric complex comprising two obligatory GluN1 subunits and two GluN2 subunits (20). Notably, the N-terminal domain (NTD) of GluN1 serves as the primary epitope for most anti-NMDAR autoantibodies identified in these patients (21). In 2019, building upon previous research, Jones et al. (22) hypothesized that intact receptor complexes play a critical role in autoimmune disorders. They actively immunized C57BL/6 adult mice with liposome-encapsulated NMDA receptor tetramers in native conformation (holoreceptors) assembled from GluN1 and GluN2B subunits. This approach successfully induced NMDAR antibodies and elicited clinical manifestations of anti-NMDAR encephalitis, including anxiety, seizures, and social deficits. Notably, histopathological analysis revealed significant inflammatory responses in the hippocampal region with characteristic tissue alterations. The experimental protocol comprised three sequential phases: first, HEK293 cells were transfected with NMDAR subunits to express GluN1 protein, followed by cell fixation, antibody incubation, washing, and affinity chromatography purification to obtain intact NMDAR receptor proteins; second, lipid components were dissolved in organic solvents and subjected to reduced-pressure rotary evaporation to form lipid thin films, which were then hydrated, filtered, and dialyzed to prepare liposomes that were subsequently mixed with purified receptor proteins at an optimized ratio and incubated followed by centrifugation to obtain NMDAR-proteoliposome complexes; third, experimental group mice received subcutaneous injections of high-purity NMDAR-proteoliposomes with booster immunization after 2 weeks, while control groups were administered equivalent volumes of blank liposomes or saline, and model validation was performed through systematic behavioral assessments including locomotor activity monitoring and seizure observation, histopathological analysis of hippocampal neuroinflammation, and immunohistochemical and Western blot detection of NMDAR expression levels, with results showing that the experimental group exhibited characteristic hyperactive behavior, significantly increased seizure frequency, and marked neuroinflammatory responses with immune cell infiltration in hippocampal regions as demonstrated by histological examination. The findings of this study demonstrate that active immunization with intact NMDA receptor proteins in mice induces disease characteristics closely resembling the core features of human anti-NMDAR encephalitis, including the production of pathogenic anti-GluN1 autoantibodies. The research demonstrates that T cells play a pivotal initiating role in the pathogenesis of NMDAR encephalitis: Native-configured NMDAR tetramers require processing by antigen-presenting cells (APCs) and subsequent MHC-II-mediated activation of CD4+ T cells, which then drive B cell activation and pathogenic antibody production, ultimately triggering neuroinflammatory responses that fully recapitulate the disease phenotype.

### 2.2 Immunological model targeting GluN1 subunit-specific peptide

According to published literature, the surface dynamics of NMDARs not only regulate long-term potentiation (LTP) at glutamatergic synapses through channel activity per se, but also contribute to long-term memory formation in the CA1 region. The trafficking of NMDARs in the dorsal hippocampal CA1 region represents a crucial cellular mechanism underlying glutamatergic synaptic plasticity, with specific NMDAR components involved in associative memory in adult rodents being modulated by estradiol (23). In 2012, Gleichman et al. (24) employed a cellular immunization model to validate GluN1 subunit peptides. Through standardized modification of patient antibody binding in transfected cells, their study identified and demonstrated that the N368/G369 region of GluN1 plays a critical role in autoimmune responses associated with anti-NMDAR encephalitis, as evidenced by analysis of engineered N-terminal GluN1 mutants. The experimental procedures were performed as follows (1): HEK293 cell transfection: HEK293 cells were transfected using the calcium phosphate method, where cells were cultured in transfection medium containing a defined ratio of glutamate receptor-specific DNA (2); Cellular immunoassay: Transfected HEK293 cells were stained, fixed, washed, and blocked, then co-incubated overnight in PBS with commercial NMDAR antibodies and patient cerebrospinal fluid (CSF), followed by validation using C-terminal GluN2A/2B antibodies (3); Site-directed and large-scale mutagenesis: PCR-based mutagenesis was performed using commercial kits according to the manufacturer’s protocols to generate large fragment deletions in GluN1. This approach ultimately identified receptor domains that simultaneously affected both antibody binding and receptor function, while also revealing receptor subdomains exhibiting variable influences on antibody binding. The patient-derived anti-GluN1 antibodies specifically bind to the extracellular amino-terminal domain (ATD) of the GluN1 subunit, inducing conformational changes (18), which subsequently lead to NMDAR clustering and clathrin-dependent internalization. Clinical studies further demonstrate a significant positive correlation between anti-GluN1 antibody titers in serum/cerebrospinal fluid and the degree of receptor internalization. These findings provide both a diagnostic biomarker for anti-NMDAR encephalitis and a molecular basis for developing ATD-targeted blocking therapies (e.g., against N368/G369 epitopes) (5).

Based on the findings of Gleichman et al. in 2020, Wagnon et al. (25) designed the GluN1359–378 peptide derived from a conserved epitope of human GRIN1 (P35439). By using complete Freund’s adjuvant (CFA) to enhance antigen immunogenicity, break immune tolerance, and promote neuroinflammation (26), they successfully induced an autoimmune response mimicking anti-NMDAR encephalitis. This model recapitulates the core pathological mechanisms of B cell-mediated neuroinflammation, providing an important tool for related research. Therefore, the experimental protocol utilized 8-week-old male C57BL/6J mice, in which autoimmune responses were induced by subcutaneous injection of an emulsified mixture (1:1 volume ratio) containing 200 μg of peptide (either GluN1_359–378_ immunogenic peptide or GluN1_168–187_ control peptide) with complete Freund’s adjuvant (CFA) supplemented with 600 μg of heat-killed Mycobacterium tuberculosis H37Ra, while the control group received 200 μl of physiological saline instead of peptide. Four 50-μl injections (total 200 μl) were administered subcutaneously at shoulders and limb regions. All mice received intraperitoneal injections of 200 ng/200 μl Bordetella pertussis toxin (PTX) both at immunization and 48 hours post-immunization. Model validation was performed through behavioral assessments, quantification of NMDAR-IgG titers, and immunohistochemical analyses, demonstrating that GluN1-induced autoimmune responses resulted in spatial memory deficits and anxiety-like behaviors, while further confirming the predominant involvement of B cell-mediated mechanisms in anti-NMDAR encephalitis pathogenesis.

Similarly, in 2021, Ding et al. (10) actively immunized C57BL/6 mice with peptides from the amino-terminal domain (ATD) of the GluN1 subunit (GluN1356-385). Ten-week-old female C57BL/6 mice were subcutaneously immunized in the dorsal region with 200 μg emulsions containing various GluN1 extracellular peptides emulsified with an equal volume of complete Freund’s adjuvant (CFA) containing H37Ra, resulting in a final peptide concentration of 1 mg/mL. Booster immunizations with the peptide emulsion were administered at 4 and 8 weeks following the primary immunization. Control mice were injected with an emulsified mixture of CFA and equal volume of phosphate-buffered saline (PBS). All mice were intraperitoneally injected with 200 ng pertussis toxin both on the day of the final immunization and 48 hours thereafter. To monitor antibody titers, cerebrospinal fluid and serum samples were collected from three mice at biweekly intervals, and behavioral assessments were conducted at 12 weeks post-primary immunization. The results demonstrated that immunization with the GluN1356–385 peptide targeting the amino-terminal domain (ATD) of GluN1 effectively induced pathogenic anti-GluN1 antibodies and elicited characteristic symptoms of anti-NMDA receptor encephalitis (10), including cognitive dysfunction, social interaction deficits, and memory impairment (7, 10, 27). Postmortem examination of brain sections revealed predominant IgG deposition in the hippocampus and cerebellum (7), with subsequent specific detection showing significant reductions in both synaptic and extrasynaptic GluN1 expression (13), accompanied by markedly decreased NMDAR cluster density in hippocampal regions, which collectively confirmed substantial loss of synaptic NMDARs. These findings provide compelling evidence for the pathogenic role of anti-GluN1 antibodies in NMDAR dysfunction and associated neuropsychiatric manifestations.

The clinical manifestations of anti-NMDAR encephalitis exhibit distinct characteristics between pediatric and adult populations. Pediatric patients predominantly present with neurological symptoms, such as epileptic seizures or behavioral disturbances (28), whereas psychiatric and behavioral manifestations tend to be less typical in this demographic (5, 7, 29, 30). Therefore, in 2023, He et al. (7) established an active immunization model in juvenile mice to investigate the clinical manifestations and mechanisms of anti-NMDAR encephalitis in children, building upon the methodology described by Ding et al. They immunized 3-week-old female juvenile mice through subcutaneous dorsal injections of GluN1 subunit peptides followed by booster immunizations. This model successfully recapitulated the clinical manifestations of human anti-NMDAR encephalitis in behavioral tests, thereby providing a juvenile mouse model for exploring the pathological mechanisms of autoimmune encephalitis in children. The immunized mice exhibited significant cognitive and social deficits, though no marked differences were observed in anxiety-like behaviors or motor functions. Electrophysiological examinations revealed that specific antibodies in the experimental group bound to NMDARs on postsynaptic membranes, resulting in reduced receptor density and impaired functionality, which led to more severe and widespread long-term potentiation (LTP) deficits. Following pharmacologically induced seizures, the experimental group demonstrated more prolonged and severe status epilepticus compared to controls (7).

In the same year, to compare the effects of different peptide administration routes and injection sites on the mouse model, Linnoila et al. (13) conducted subcutaneous injections of equal peptide doses at dorsal sites (both with and without boosters immunizations) and ventral abdominal sites. Their study investigated the time course of antibody production and clinical manifestations induced by each administration method. The results demonstrated that the abdominal injection group developed antibodies earliest, achieving serum anti-NMDAR antibody titers comparable to those of the dorsal injection groups by week 4 post-immunization. The peak serum antibody titers in mice were independent of the injection methods, uniformly peaking at week 6. However, cerebrospinal fluid antibody titers reached their peak earlier in the abdominal subcutaneous immunization group and dorsal booster immunization group (week 6), compared with the dorsal non-booster group (week 8). Flow cytometry and histological analyses (13) revealed lymphocyte and plasma cell infiltration in the brains of immunized mice, with the timing and duration of immune cell infiltration correlating with subcutaneous injection sites. Cytokine levels also exhibited significant temporal fluctuations, potentially reflecting the progression of immune responses and inflammatory processes in mice. This study provides crucial reference data for future active immunization mouse model development, offering guidance on optimal injection methods and sites, while enabling researchers to design optimized experimental protocols based on antibody kinetic profiles and peak titer timelines.

In 2024, Xia et al. (27) established an animal model by subcutaneously injecting specific peptides into the dorsal region of mice, followed by booster immunizations with peptide emulsions at 4 and 8 weeks post-primary immunization, while control groups received equivalent volumes of buffer solution. Behavioral assessments were conducted alongside monitoring of antibody titers and specificity. This study represents the first demonstration of significant NLRP3 signaling pathway hyperactivation in an anti-NMDAR encephalitis animal model, which was closely associated with microglial activation. Notably, MCC950 (a selective NLRP3 inflammasome inhibitor) administration (31) effectively attenuated neuroinflammation, ameliorated cognitive deficits, and exerted neuroprotective effects in anti-NMDAR encephalitis, thereby providing novel therapeutic insights for this condition.

### 2.3 Immunological model of HSV infection

Current evidence confirms that tumors (particularly teratomas (3, 32)) and infections (mainly HSV (33)) represent the two primary triggers for anti-NMDAR encephalitis. While no animal model exists for teratoma-induced anti-NMDAR encephalitis, infection-triggered active immunization has consequently emerged as a key research focus for mechanistic studies (34). Anti-NMDAR antibodies are frequently detected in herpes simplex virus encephalitis (HSE) patients, where they alter levels of NMDARs and other synaptic proteins (35). Notably, post-HSE recurrence shows high propensity to induce anti-NMDAR encephalitis (36), with some HSE patients developing autoimmune encephalitis symptoms within 3 months after completing acyclovir (ACV) treatment (33) (a nucleoside analogue that inhibits viral DNA polymerase upon cellular activation (37)). This mechanistic link prompted Linnoila et al. (2019) (14) to successfully establish an active immunization mouse model of anti-NMDAR encephalitis through intranasal HSV inoculation. Six age- and size-matched female BALB/c mice received intranasal inoculation with 0.5–1 × 10^6 PFU of HSV-1 (strain 17syn+) (38), concurrently administering intraperitoneal ACV antiviral therapy (20 mg/kg, twice daily). Two weeks post-inoculation, the incidence of anti-NMDAR encephalitis was evaluated. The results demonstrated that 66.7% of the experimental mice developed serum anti-NMDAR antibodies. Successful model establishment was evidenced by (1): significant reduction of NMDARs in hippocampal postsynaptic membranes (2); decreased hippocampal NMDAR protein levels, and (3) the observed NMDAR depletion pattern mirroring that caused by patient-derived antibodies. This study provides a valuable reference for investigating infection-triggered autoimmune encephalitis and facilitates exploration of upstream antigen-antibody interaction mechanisms.

## 3 Active immunization animal models for anti-NMDAR encephalitis: advantages, limitations, and feasibility

The NMDA receptor full-length protein active immunization model developed by Jones et al. represents the pioneering approach in active immunization models, establishing a foundation for subsequent animal models of anti-NMDAR encephalitis and providing an effective platform for investigating its pathophysiological mechanisms and developing novel therapeutic strategies. They successfully established an anti-NMDAR encephalitis animal model using native-configured NMDAR tetrameric antigens. This model activates a CD4+ T cell-dependent B cell immune response, induces production of GluN1 subunit-specific antibodies, and triggers neuroinflammatory reactions, thereby fully recapitulating the entire disease progression from immune activation to clinical manifestations (including behavioral abnormalities, motor dysfunction, and spontaneous epileptic seizures). However, the full-length protein immunization technique presents significant technical challenges primarily due to the structural complexity of intact NMDA receptors (20), their susceptibility to conformational changes or degradation during *in vitro* expression and purification processes (39), and the difficulty in maintaining native conformation and antigenicity, resulting in insufficient specificity despite requiring optimized protein stabilization protocols (7, 22). Notably, while T-cell involvement was confirmed in this full-length protein immunization model, current evidence predominantly supports the central role of B cells in anti-NMDAR encephalitis pathogenesis (40), warranting further investigation to validate this hypothesis.

Active immunization induced by GluN1 subunit peptides is based on the principle that polypeptide fragments can generate autoantibodies targeting glutamate receptors, reduce seizure thresholds, and induce behavioral alterations in mice. Successful establishment of active immunization using GluN1 subunit peptides has demonstrated that the GluN1356–385 peptide targeting the ATD of GluN1 effectively induces pathogenic anti-GluN1 antibodies in mice. These antibodies recognize epitopes similar to those targeted by patient-derived antibodies and recapitulate characteristic symptoms of anti-NMDAR encephalitis (10). Usually, antibody-mediated encephalitides are typically characterized by concomitant neuroinflammation and seizure activity. Unlike other neurological disorders or experimental models, these conditions provide direct evidence for synaptic dysfunction and neuronal hyperexcitability through their well-defined mechanisms involving specific antibody binding to synaptic receptors and proteins (41). The GluN1 peptide-induced NMDAR encephalitis model exhibits significant limitations: while linear peptides can directly activate B cells and generate high-titer antibodies, their inability to engage T cell assistance and relevant inflammatory signaling pathways prevents complete recapitulation of human disease pathology. This model fails to reproduce hallmark clinical manifestations of anti-NMDAR encephalitis (including movement disorders, psychiatric symptoms, and spontaneous seizures), while demonstrating marked deficiencies in investigating critical aspects such as disease pathogenesis, inflammatory cytokine networks, and immune cell interactions. These shortcomings substantially constrain the model’s utility for comprehensive mechanistic studies of NMDAR encephalitis.

HSV infection of the central nervous system represents a significant trigger for anti-neuronal autoimmunity (42). Current scholarship predominantly supports the view that HSV infection serves as an indirect precipitating factor for anti-NMDAR encephalitis, which may explain the relative scarcity of such models in research. The pathogenic mechanism is not attributable to direct viral neurotoxicity, but rather stems from secondary immune-mediated damage following viral infection (35, 43). This pathophysiological process involves three key interrelated mechanisms: First, molecular mimicry occurs between HSV-1 proteins (e.g., glycoprotein B) and human synaptic proteins (e.g., NMDA receptors), leading to cross-reactive autoantibody production (44). Second, viral PAMPs are recognized by microglial TLR3 receptors, triggering innate immune responses characterized by excessive IFN and proinflammatory cytokine (IL-6, TNF-α) release, blood-brain barrier disruption, and CD8+ T cell infiltration (45). Finally, a self-sustaining cycle develops through persistent inflammatory microenvironments and T cell-mediated bystander neuronal damage (46). These mechanisms, which have been experimentally validated in animal models (38, 44), collectively establish the comprehensive pathophysiological foundation for post-infectious autoimmune encephalitis, with immune-mediated injury playing the central pathogenic role. Therefore, Linnoila (14) et al. developed an HSV-induced active immunization model that accurately replicates the complete clinical progression observed in patients─from initial HSV infection to HSE and subsequent development of anti-NMDAR encephalitis. Employing identical methodologies used for detecting NMDAR antibodies in human clinical samples, serum NMDAR antibodies were successfully identified in murine blood specimens. However, their model validation relied solely on serum anti-NMDAR antibody titers as the success criterion, failing to assess CSF antibody levels, clinical-behavioral associations, or cytokine profiles. Furthermore, the inherent complexities of concurrent viral infection and antiviral therapies necessitate additional experimental validation to confirm model reliability. Notably, other viral infections - particularly Japanese encephalitis virus (JEV) - have also been documented to trigger similar NMDAR antibody-associated post-viral encephalitis (28). Additionally, HSV infection may induce encephalitis mediated by antibodies targeting other undefined neuronal surface proteins. These observations collectively underscore the need for further refinement of the model’s specificity through comprehensive characterization of antibody profiles and exclusion of confounding immune responses (47).

In current research, the active immunization model takes priority over the passive immunization model in simulating the pathophysiological processes and clinical manifestations of anti-NMDAR encephalitis, as well as investigating its pathogenesis (7). There are mainly two types of previous passive immunization models, both with significant limitations: one involves delivering exogenous anti-NMDAR antibodies derived from patients to mice via intracerebroventricular injection. Although this method has been confirmed to induce NMDA receptor internalization in the hippocampus and reduce synaptic density (48), it bypasses the blood-brain barrier through intracerebroventricular injection, interfering with the natural pathophysiological process. Additionally, it relies on pentylenetetrazol (PTZ) to induce epilepsy and cannot simulate spontaneous epilepsy phenotypes; meanwhile, the total IgG in patient serum contains non-specific antibodies, which are prone to causing mechanism confusion. Furthermore, the expression of the target antigen LGI1 is not significantly reduced, which further impairs pathological relevance (49, 50). The other is the humanized passive transfer model established by Yaqing Shu’s team in 2023 (51). Specifically, female BRGSF mice (B/T/NK cell-deficient) aged 8–10 weeks were employed in this study, which were randomly allocated into three groups receiving intraperitoneal injections: sterile RPMI 1640 medium (NC group), PBMCs from healthy volunteers (HC group), or PBMCs from affected patients (patient group). Although this model has confirmed that patient-derived peripheral blood mononuclear cells (PBMCs) can specifically induce the production of anti-GluN1 autoantibodies and elucidated the role of the IL-1β signaling pathway in blood-brain barrier disruption through the integration of humoral immunity assays, neurobehavioral evaluations, and neuropathological analyses—providing a novel experimental platform for the development of therapeutic strategies—it essentially falls into the category of passive immunization. It neither fully recapitulates the natural physiological process of autoantibody production *in vivo* nor is it without limitations in the assessment of cellular functions such as synaptic transmission efficiency and synaptic plasticity. In contrast, the active immunization model possesses three key advantages: 1) It can fully recapitulate the cascade reaction of antigen presentation and antibody production, which is more consistent with the spontaneous immune activation process of human diseases (52); 2) It can maintain the integrity of the dynamic function of the blood-brain barrier and avoid interference of exogenous interventions on the natural pathophysiological process (53); 3) It can more accurately present the synaptic pathophysiological characteristics and improve the accuracy of mechanism research (17). Crucially, given that the pathogenesis of autoimmune encephalitis remains unclear, there is an urgent need for an effective animal model that can restore the natural occurrence of the disease to explore the correlation between synaptic membrane proteins and the disease—and the active immunization model precisely meets this core requirement. Furthermore, although humanized mouse models have been widely utilized in the research of various autoimmune diseases, such as myasthenia gravis (54), idiopathic nephrotic syndrome (55), systemic lupus erythematosus (56, 57), systemic sclerosis (58, 59), and Rasmussen’s encephalitis (60), the core logic of their PBMC-based passive transfer still cannot replace the unique value of active immunization models in recapitulating the intrinsic immune responses of the disease.

## 4 Current applications and future prospects of active immunization models in anti-NMDAR encephalitis research

The anti-NMDAR encephalitis immunization model serves as a crucial tool for investigating the pathogenesis of this disease and developing therapeutic interventions, with significant advancements achieved in recent years in model construction, mechanistic elucidation, and therapeutic strategy exploration. Evaluating the comparative advantages and limitations between active and passive immunization models has become a prerequisite for future mechanistic studies. If the research objective is to investigate the direct effects of antibodies on the nervous system or their pathogenic mechanisms from production to disease manifestation *in vivo*, the active immunization model would be the preferred choice. Conversely, if the study requires precise control over antibody dosage and specificity for short-term investigations, particularly in examining the relationship between viral infections and autoimmune responses, the passive immunization model would be more suitable (**Table 1**).

**Table 1:** Comparative analysis of active vs. passive immunization models for autoimmune neurological disorders.

Comparison dimension	Active immunization model	Passive immunization model
Core Principle	Activates the host's immune system with antigens (full protein/peptide/virus) to produce endogenous antibodies	Directly injects exogenous antibodies (patient CSF/IgG) into animals
Key Techniques	- Full NMDA receptor protein immunization - GluN1 peptide immunization - HSV infection induction	Intracerebroventricular (ICV) injection of human- derived NMDAR antibodies
Pathophysiological Relevance	- Fully recapitulates antigen presentation and antibody generation cascade - Maintains dynamic BBB function - Accurately mimics synaptic pathology features (e.g., receptor internalization)	- Intervenes natural process via BBB bypass - Fails to demonstrate endogenous antibody production - Impairs synaptic transmission efficiency/plasticity
Clinical Symptom Correlation	- Recapitulates core symptoms: spontaneous seizures, motor deficits, psychiatric/behavioral abnormalities	- Cannot model spontaneous seizures (requires PTZ induction) - Poor clinical phenotype correlation
Major Limitations	- Incomplete symptoms in peptide/HSV models (e.g., lacking spontaneous seizures) - Antigen stability challenges	- Patient IgG contains mixed specific antibodies mechanistic confusion - Target antigen (e.g., LGI1) expression not reduced insufficient pathological relevance
Applicability	Studying full disease course mechanisms, therapeutic target validation, prevention strategy development	Limited to short-term antibody effect studies

As mentioned previously, the active immunization animal model for anti-NMDAR encephalitis serves to better explore the disease’s pathogenesis and the immune pathways involved, thereby facilitating clinical applications and drug development. The field of anti-NMDAR encephalitis research has developed multiple active immunization animal models, though the preparation processes of these models are generally characterized by operational complexity, high costs, and prolonged experimental durations. To address these challenges, researchers continue to refine model construction strategies by systematically evaluating key parameters including antigen types (full-length proteins, the GluN1 peptides, and HSV vectors), immunization protocols (dose and frequency), and host factors (animal age and sex). In this context, the full-length protein model utilizes native-configured NMDAR tetramers to comprehensively simulate the coordinated T-cell and B-cell immune response and the complete neuroinflammatory cascade, encompassing GluN1-specific antibody production and characteristic clinical manifestations such as behavioral abnormalities, motor deficits, and epileptic seizures. However, due to its technical complexity, this model is particularly suited for mechanistic studies requiring high-fidelity pathological replication. While HSV-induced models can mimic virus-triggered autoimmune processes, their lack of specificity for anti-NMDAR encephalitis and limited reported cases in current research restrict their application in pathogenesis studies. In contrast, peptide models employ linear antigens delivered via abdominal subcutaneous injection to rapidly activate B-cells and induce anti-NMDAR antibody production. Although these models lack T-cell involvement, experiments demonstrate detectable immune responses and behavioral changes in early stages, making them ideal for short-term studies without requiring booster immunizations. When utilizing dorsal subcutaneous booster injections, antibody titers and immune response intensity are significantly enhanced, facilitating establishment of stable long-term pathological models. These models demonstrate significant advantages characterized by straightforward operational procedures requiring only single injections to achieve phenotypic outcomes within two to four weeks, reduced experimental durations, and cost efficiency, rendering them particularly suitable for application-oriented investigations including antibody profiling and pharmaceutical screening. Consequently, investigators can optimize immunization strategies by selecting either accelerated protocols for short-term observational studies or enhanced regimens for long-term pathological maintenance, with the choice being determined by specific research goals. (**Table 2**). Although circumventing exogenous antibody interference, this active immunization model exhibits significant limitations: it exclusively targets linear epitopes (61) (lacking native conformation-dependent epitopes), requires potent adjuvants (complete Freund’s adjuvant combined with pertussis toxin) that induce non-specific inflammation, and fails to reliably replicate core phenotypes such as spontaneous epilepsy (e.g., only a subset of animals developed convulsions in Ding et al.’s study (10)). These deficiencies may stem from endogenous antibodies primarily driving receptor internalization (distinct from acute synaptic inhibition mechanisms), undetected subclinical seizures due to unmonitored EEG, insufficient observation periods to capture delayed epilepsy onset, and absence of significant complement activation or pro-epileptogenic cytokine induction (**Table 3**). Future research directions should incorporate cutting-edge methodologies such as *in vivo* imaging and single-cell sequencing to enable dynamic monitoring of synaptic pathology and neuroinflammatory cascades. Despite substantial progress in clinical characterization, the field urgently requires standardized diagnostic criteria encompassing both clinical and immunological parameters, deeper mechanistic insights into neuroimmunological interactions, and identification of reliable biomarkers correlating with disease progression and therapeutic outcomes, all of which are critical for developing novel treatment modalities, optimizing therapeutic efficacy, accelerating clinical recovery, and ultimately improving patient prognosis (28).

**Table 2:** Comparison of injection methods in NMDAR encephalitis models.

Injection method	Advantages	Disadvantages
Ventral Subcutaneous Injection	- Rapid induction of NMDAR-related pathology and behavioral features - Early observation of immune response and behavioral changes (e.g., at 4 weeks) - Suitable for short-term studies	- Requires frequent monitoring to capture early changes - Long-term effects may diminish; not suitable for long-term studies
Dorsal Subcutaneous Injection	- Minimal physiological disturbance to mice - Suitable for long-term studies with stable pathological changes	- Requires booster injections to observe significant changes, increasing experimental complexity - Slower immune response; limits use in rapid studies
Dorsal Subcutaneous Boosted Injection	- Booster injections enhance antibody production and immune response intensity - Maintains or enhances long-term pathology and behavioral changes	- Booster injections increase stress and discomfort in mice - Requires precise timing and additional resources

Ventral subcutaneous injection: Administered at bilateral axillary and inguinal regions; Dorsal subsutaneous boosted injection: Administered at dorsal subcutaneous region.

**Table 3:** Comparison of three active immunization animal models for anti-NMDAR encephalitis.

Model type	Advantages	Disadvantages
Immunological Model Based on Full-Length NMDA Receptor Protein	- Pioneering active immunization protocol - Successfully replicates behavioral abnormalities, movement disorders, seizures, and other clinical manifestations - Detects serum antibodies targeting GluN1 subunit - Provides platform for mechanism research and therapeutic development	- Technically challenging: complex receptor structure - Conformational changes/degradation during expression/purification - Difficulties maintaining native conformation/antigenicity - Insufficient specificity even after optimization
Immunological Model Targeting GluN1 Subunit-Specific Peptide	- Simple materials and convenient preparation - Confirms pathogenicity of GluN1356-3speptide-induced antibodies - Induces GluN1 antibodies in mice with similar characteristics to patient antibodies - Recapitulates typical anti-NMDAR encephalitis symptoms - Reveals direct mechanism of synaptic dysfunction	- Lacks key manifestations: movement disorders, psychiatric symptoms, spontaneous seizures - Incomplete understanding of pathogenesis, inflammatory cascades, and mechanisms - Requires handling antiviral treatment complexities - Limited specificity (may induce other antibody-mediated encephalitis) - Requires multi-parameter validation
Immunological Model of HSV Infection	- Fully replicates clinical progression from HSV infection to secondary encephalitis - Confirms HSV as potent autoimmune trigger - Mechanism involves post-viral immune injury, enabling study of virus- autoimmunity relationships - Facilitates exploration of upstream antigen-antibody binding mechanisms	- Relies solely on serum antibody titers for model validation - Lacks evaluation of CSF antibodies and clinical manifestations - Fails to demonstrate in vivo autoantibody production process

## 5 Discussion

Anti-NMDAR encephalitis is a rare autoimmune disorder characterized by antibodies targeting the GluN1 subunit of NMDAR. These antibodies attack central neuronal synaptic NMDA-type glutamate receptors, leading to a range of clinical manifestations (1). However, the pathogenesis remains complex and currently lacks a unified consensus. Mouse models have played an instrumental role in advancing understanding of disease mechanisms, identifying novel therapeutic targets, and facilitating translational research. This review focuses on the established methodologies for constructing active immunization models of anti-NMDAR encephalitis, critically analyzes the advantages and limitations of each approach, and evaluates the current applications and future prospects of these models. The findings provide valuable references for future mechanistic studies on anti-NMDAR encephalitis. In current cultured neuron models, antibodies reduce NMDAR density without affecting synaptic number. In NMDAR mouse models, the mechanisms underlying NMDAR internalization, potential disruption of other NMDAR-interacting proteins, or impaired synaptic plasticity remain uncharacterized. While these mouse models exhibit phenotypes resembling clinical manifestations of the human disease, the severity of symptoms and their potential reversibility remain undetermined (28), with no published studies addressing these questions. In summary, the optimization of active immunization models for anti-NMDAR encephalitis constitutes an indispensable component of future mechanistic investigations. Refinement of animal model systems may involve genetic engineering technologies (e.g., CRISPR/Cas9 (62, 63)) or incorporation of human immune system components (e.g., humanized mouse models (51, 64)) to better recapitulate the pathological mechanisms of human anti-NMDAR encephalitis, while parallel development of tumor-associated models (e.g., ovarian teratomas (65)) is essential for elucidating tumor-disease interactions. Further improvements in model fidelity can be achieved through utilization of monoclonal antibodies (21, 66) or antibody fragments that more closely resemble patient-derived anti-NMDAR antibodies. Comprehensive investigation of immunopathological mechanisms should extend beyond characterizing antibody-NMDAR interactions to include the roles of T cells, B cells, microglia, and other immune mediators (34). Concurrent optimization efforts must focus on developing precise diagnostic tools, including identification of biomarkers for early diagnosis or disease progression prediction, as well as non-invasive detection methods utilizing blood or cerebrospinal fluid. Therapeutic development should prioritize targeted interventions against NMDAR antibodies (e.g., ART5803 binds GluN1-NTD while preserving normal NMDAR activity and localization (15)) or associated immune responses, encompassing evaluation of immunomodulatory agents and cellular therapies. Through sustained research endeavors, the development of more sophisticated murine models is expected to facilitate a deeper understanding of the pathological mechanisms underlying anti-NMDAR encephalitis, enable the creation of more effective therapeutic approaches, and ultimately lead to significant improvements in patient quality of life.
